# Statin Treatment in Specific Patient Groups: Role for Improved Cardiovascular Risk Markers

**DOI:** 10.3390/jcm9113748

**Published:** 2020-11-21

**Authors:** Alyssa M. B. White, Hillary R. Mishcon, John L. Redwanski, Ronald D. Hills

**Affiliations:** 1 Department of Pharmaceutical Sciences and Administration, University of New England, Portland, ME 04103, USA; awhite21@une.edu (A.M.B.W.); hmishcon@une.edu (H.R.M.); 2 Department of Pharmacy Practice, School of Pharmacy, University of New England, Portland, ME 04103, USA; jredwanski@gmail.com

**Keywords:** cardiovascular disease, statins, primary prevention, geriatrics, risk biomarkers, cardiovascular risk calculators, low-density lipoprotein cholesterol, lipoprotein subfractions, coronary artery calcification, coenzyme Q

## Abstract

Ample evidence supports the use of statin therapy for secondary prevention in patients with a history of atherosclerotic cardiovascular disease (ASCVD), but evidence is wanting in the case of primary prevention, low-risk individuals, and elderly adults 65+. Statins are effective in lowering low-density lipoprotein (LDL), which has long been a target for treatment decisions. We discuss the weakening dependence between cholesterol levels and mortality as a function of age and highlight recent findings on lipoprotein subfractions and other superior markers of ASCVD risk. The efficacy of statins is compared for distinct subsets of patients based on age, diabetes, ASCVD, and coronary artery calcium (CAC) status. Most cardiovascular risk calculators heavily weight age and overestimate one’s absolute risk of ASCVD, particularly in very old adults. Improvements in risk assessment enable the identification of specific patient populations that benefit most from statin treatment. Derisking is particularly important for adults over 75, in whom treatment benefits are reduced and adverse musculoskeletal effects are amplified. The CAC score stratifies the benefit effect size obtainable with statins, and forms of coenzyme Q are discussed for improving patient outcomes. Robust risk estimator tools and personalized, evidence-based approaches are needed to optimally reduce cardiovascular events and mortality rates through administration of cholesterol-lowering medications.

## 1. Introduction

Atherosclerotic cardiovascular disease (ASCVD), a leading cause of morbidity, accounts for 1 in 3 deaths in U.S. adults [1]. Statin drugs, which inhibit the body’s endogenous synthesis of cholesterol, have become a mainstay in the primary prevention and treatment of existing ASCVD and are now one of the most commonly prescribed drugs in the world [2]. From 2002 to 2013, statin use among US adults 40 years and older almost doubled, from 22 to 39 million adults, or 28% of the population [3]. The Medical Expenditure Panel Survey found that statin use for primary ASCVD prevention in adults 80 years and older increased four-fold, from 9% in 1999 to 34% in 2012 [4]. The 2013 American College of Cardiology/American Heart Association (ACC/AHA) guidelines for the management of blood cholesterol identified 13 million Americans as newly eligible for consideration statin therapy based on a 10-year ASCVD risk ≥7.5%, which is a lower threshold compared to other international guidelines [5,6]. Given the potential adverse effects of statin use, recent meta- and post-hoc analyses have attempted to address whether available clinical evidence supports such widespread use, particularly in low-risk individuals [7].

Early statin trials excluded elderly patients, and less evidence exists overall for the efficacy of statins in older adults [8]. While no randomized controlled trials (RCTs) have been conducted using statins in patients older than 80 years at baseline, the independent STAREE (NCT02099123) and PREVENTABLE (NCT04262206) trials are currently assessing high intensity statin therapy compared to placebo in adults 70+ and 75+, respectively. Most meta-analyses of statin use to date have included participants for both primary and secondary prevention, with fewer adults over 65 being represented [2,9,10]. To balance the potential benefits and harms from statin therapy, efficacy must be compared across different patient groups of varying cardiovascular risk [11]. In this review, we discuss the role of cholesterol in mortality and the efficacy of statins as a function of age and other determinants of ASCVD risk. Patients with no history of ASCVD account for most of the recent increase in statin use [2], for which the decision to treat has rested heavily on the accuracy of evolving ASCVD risk calculators [12]. The totality of evidence reveals that the benefit effect size obtained from statin treatment is linked to patient age as well as the presence of strong ASCVD risk factors. While the relationship between serum cholesterol and health outcomes is complicated, a new assortment of cardiovascular risk markers has emerged for determining who best benefits from lipid-lowering treatment. These observations should serve to guide the development of evidence-based recommendations for clinical practice.

## 2. Potential for Adverse Effects

The most common adverse effect of statins are musculoskeletal events including myopathy, myalgias, muscle weakness, arthropathies, and, rarely, rhabdomyolysis [13,14,15]. Estimates of the number needed to harm (NNH) in retrospective studies have ranged from 19 to 47 for musculoskeletal disorders [7,16,17], and statins markedly increase the risk of myopathy [odds ratio (OR): 2.63, 95% confidence interval (CI): 1.50–4.61] [18]. A recent analysis considered 60,455 military adults in the TRICARE medical program and propensity matched statin users with nonusers [19]. The statin users (72% used simvastatin) were observed to have a higher likelihood of back disorder diagnosis (OR: 1.27, 95% CI: 1.19–1.36, NNH = 17 at 4 years). The odds ratio increased to 1.47 when the cohort was restricted to users of statins for more than two years or to high-intensity statin users vs. nonusers. A case of rhabdomyolysis was recently reported when canagliflozin was administered to a 5-year rosuvastatin user [20]. Canagliflozin is a diabetes drug and transport inhibitor that enhances statin absorption; the patient’s plasma rosuvastatin concentration was 15-fold higher than expected for a 40 mg dose, and the case resolved once the two drugs were stopped. While high-intensity statins can be avoided to reduce the severity of adverse effects, muscle symptoms are also reported with moderate-intensity therapy [14]. Muscle weakness and pain pose a particular problem for the elderly, in whom statins might contribute to deconditioning, frailty, and falls [21].

Analysis of medical claims data for the entire Austrian population (*n* = 7,897,449) recently revealed a dose-dependent response in the association of statin use with osteoporosis [22]. Low-dose treatment (10 mg/day) with lovastatin, pravastatin, simvastatin, or rosuvastatin was observed to decrease risk. However, for each of six statins osteoporosis diagnosis increased with dosage and was overrepresented in 60 and 80 mg/d simvastatin [odds ratios (ORs): 1.64 and 3.30], 40 mg/d rosuvastatin (OR: 2.04, 95% CI: 1.31–3.18), and 20, 40, 60 and 80 mg/d atorvastatin (ORs: 1.35, 1.78, 2.12 and 3.14). Interestingly, elevated osteoporosis risk was primarily observed for adults 60 and younger and was more pronounced in females. Sex hormones, which are made from cholesterol, decline with age and correlate with osteoporosis, and statins have been shown to reduce plasma levels of estradiol and testosterone [22].

As noted in a 2012 Food and Drug Administration warning, statin use carries a modest increase in the relative risk (RR) of new-onset type 2 diabetes [7,23]. Individual statins have been found to increase blood glucose, reduce insulin secretion and/or decrease insulin sensitivity [24,25,26]. Meta-analysis of 20 observational studies found that long-term statin use increases the risk of developing incident diabetes relative to nonusers (RR: 1.44, 95% CI: 1.31–1.58) [27]. High potency statins had the highest risk (rosuvastatin, RR: 1.61, 95% CI: 1.30−1.98). A 14-year retrospective study of 471,250 elderly patients in Ontario calculated the 1-year NNH for atorvastatin, rosuvastatin, and simvastatin (NNH: 172, 210, and 363, respectively) [24]. A Women’s Health Initiative study of postmenopausal women (ages 50-79) estimated an NNH = 44 (95% CI: 35−60) for an additional case of diabetes over three years of statin therapy [18]. While the molecular mechanism of diabetogenic action remains unclear [26], new data have shown the hydrophilic statins (rosuvastatin and pravastatin) to increase insulin resistance to a greater degree than lipophilic statins [28].

## 3. Cholesterol Levels and Clinical Outcomes

### 3.1. Low-Density Lipoprotein and Total Cholesterol

Plasma low-density lipoprotein cholesterol (LDL-C) has been a principal target of cholesterol-lowering therapy for the past three decades, and statins are effective in lowering LDL-C to reduce ASCVD. Data on the relationship between LDL-C and all-cause and cardiovascular mortality rates, however, reveal a paradoxical association. Ravnskov et al. conducted a systematic review of 19 clinical studies involving 68,094 patients 60 years or older [29]. In 16 different cohorts accounting for 92% of participants, an inverse association was found between LDL and all-cause mortality, with a mean hazard ratio (HR) = 0.54 for the highest vs. lowest quartile LDL-C. Studies reporting cardiovascular mortality did not find an overall association with LDL [29], except for one study which measured deaths in each LDL quartile, observing an almost U-shaped dependence with the highest risk in the lowest LDL quartile [30]. More recently, a population study of 262,391 Korean adults over 40 who did not use statins revealed that changes in total cholesterol (TC) within a two-year period increase the risk of subsequent all-cause mortality relative to those who maintained levels in the second tertile [31]. Among the elderly, frailty and poor health status likely contribute to increased risk of death in those with low cholesterol. TC levels are known to decline in the last stages of life in statin users and nonusers [32,33], and low cholesterol can be a marker of severe diseases such as cancer [34].

Using LDL-C as an ASCVD predictor is problematic for a number of reasons. LDL was dropped from the 2008 Framingham calculator for measuring one’s 10-year ASCVD risk because it did not improve the model fit [35]. While laboratory tests now exist for direct measurement of LDL plasma concentration, LDL-C is traditionally calculated using the Friedewald equation (Equation (1)) developed in the 1960s, which is a function of high-density lipoprotein cholesterol (HDL-C) [36]:

(1) $${\mathrm{LDL}-C}_{F}\;(\mathrm{mg}/\mathrm{dL})\;=\mathrm{TC}-\mathrm{HDL}-\left(\frac{\mathrm{triglycerides}}{5}\right)$$

Calculated LDL-C measurements rely on the assumption that the ratio of triglycerides to very low-density lipoprotein (VLDL) is five, but this ratio actually varies between individuals from 2 to 15 [37]. An adaptable equation, LDL-C_N_, has since been developed that estimates an individual’s triglycerides:VLDL ratio in order to better approximate directly measured LDL in patients with triglycerides < 400 mg/dL [37]. LDL-C_N_ has been shown to be more accurate (range, 87–94%) than Friedewald (range, 71–93%) in both fasting and non-fasting samples [38]. In settings where LDL < 70 mg/dL and triglycerides were 200–399 mg/dL, LDL-C_N_ is far more accurate (range, 82–94%) than Friedewald (range, 37–96%).

### 3.2. Lipoprotein Subfractions

While the bulk of clinical studies have employed traditionally calculated LDL-C (mass/volume), the number of lipoprotein particles correlates better with ASCVD than the overall mass concentration [39]. A single apolipoprotein B is responsible for organizing the lipids within an LDL particle, making plasma levels of apolipoprotein B indicative of LDL particle number [40,41]. In 11,186 JUPITER trial participants with LDL-C < 130 mg/dL, baseline apolipoprotein B and LDL particle number but not LDL-C were observed to correlate with first major adverse cardiovascular event (MACE), though a correlation was observed with on-treatment LDL-C levels in the statin users [42]. Similarly, baseline and on-statin HDL particle number (HDL-P) was shown to be the strongest of four HDL-related predictors for ASCVD [43]. In the Dallas Heart Study, HDL-P but not HDL-C was found to inversely associate with coronary heart disease (CHD) in Black participants [44]. Measuring subfractions of LDL particles based on size is yet another means of getting at particle abundance. For individuals with the same LDL-C, the amount of small dense LDL (sdLDL) particles in proportion to large, buoyant LDL (lbLDL) correlates positively with the number of particles and one’s ASCVD risk [45,46]. Genetic variation in sdLDL metabolism has been associated with CHD, and sdLDL levels are a strong predictor of CHD (HR: 1.51 highest/lowest quartile, 95% CI: 1.21–1.88), even when LDL-C levels are low [47].

## 4. Accuracy of Cardiovascular Risk Calculators: The Pooled Cohort Equations

Available instruments for estimating a patient’s 10-year risk of first cardiovascular event vary widely in their predictions and application, and are often insufficiently reported [6,48,49]. The Framingham total cardiovascular risk, last updated in 2008, is still recommended by the Canadian Cardiovascular Society [40], which recommends pharmacotherapy for individuals with a risk score ≥ 10% and LDL-C ≥ 3.5 mmol/L. Since the 2013 update, the ACC/AHA guidelines have de-emphasized LDL targets for cholesterol therapy, favoring a reliance on risk estimation in low to intermediate-risk individuals. In conjunction with development of the 2013 ACC/AHA guidelines, a new heretofore unpublished estimator tool, known as the pooled cohort equations (PCEs), was created that replaced the Framingham score in the U.S. as the only calculator derived from U.S. populations [50,51,52].

Independent analyses were quick to show that the PCEs overestimate an individuals’ risk up to two or more times the actual rate of ASCVD events observed in different validation cohorts [53,54,55,56,57,58,59]. In so doing, the 2013 ACC/AHA guidelines made nearly half of U.S. adults and up to 87% of men aged 60–75 eligible for statin treatment [12]. Importantly, the PCEs and other risk calculators heavily weigh age as a determining factor [6]. Most older patients, especially those over 75, exceed the 7.5% 10-year ASCVD risk threshold for treatment despite the absence of dyslipidemia, diabetes, hypertension, or smoking [60,61]. Based on the totality of evidence, the U.S. Preventive Services Task Force has recommended raising the 10-year risk threshold for moderate-intensity statin treatment from 7.5% to 10% for adults aged 40–75 with no history of ASCVD [61].

Various problems have been noted with the derivation of the 2013 PCEs. The patient data set underrepresented Black adults, and model overfitting resulted in extreme risk estimates relative to white adults with the same risk factors [58]. The data set also did not reflect modern U.S. society, with most patient cohorts accrued from the 1960s through 1980s in addition to the original Framingham study of 1948 [54]. Yadlowsky et al. recently completed a rigorous statistical analysis of the 2013 PCEs and found that they violate the Cox proportional hazards assumption [58]. Using updated statistical methods and incorporating data from modern cohorts, the authors were able to derive a revised set of equations, which resulted in 12 million fewer U.S. adults exceeding the 7.5% risk treatment threshold. Analogously, a New Zealand population study using the PREDICT cohort of primary care patients aged 30–74 successfully replaced the PCE equations by adding variables for socioeconomic deprivation and self-identified ethnicity, which were both shown to be independent predictors [59]. The equation risk predictions closely matched the observed five-year rates of cardiovascular disease. However, the same equations progressively lost accuracy when applied to very old adults over 75 [62]. The adoption of updated risk calculators based on relevant cohort data will be needed in future clinical practice to maximize the benefits and minimize adverse events associated with statin therapy.

There are other areas for improvement in the PCEs, such as including chronic kidney disease, a high-risk condition [51]. Central obesity is also a well-known contributing factor in ASCVD, yet it is not accounted for in the Framingham or 2013 ACC/AHA risk calculators [52]. While body mass index (BMI) did not improve model performance in the Framingham calculator [35], waist-to-hip ratio and the conicity index, also a function of waist circumference, have since emerged as superior predictors of ASCVD risk across nationalities, especially in women [63,64,65]. Lastly, the risk calculators use TC as an independent variable, but mortality is a non-monotonic function of TC whose shape varies with sex and age [66,67]. A recent prospective study of 12,815,006 Korean adults found U-shaped curves of varying slope for all-cause mortality as a function of TC across 12 different age and sex groups [67]. In each of the six age groups spanning 18–99 years, mortality decreased steeply (13–34%) for each mmol/L (39 mg/dL) increase in the TC range < 200 mg/dL. For TC ≥ 200 mg/dL, there was a more modest increase in mortality of 3–14% per mmol/L. Associations between mortality and TC were steepest in age groups 35–44, 45–54, and 55–64, especially in men. These findings, together with the overestimation of ASCVD risk, help illustrate the shortcomings of relying on simplistic risk calculators to make treatment decisions [6,48,59].

## 5. Methodology

Given the interaction between cholesterol levels, mortality, and age, we reviewed the current literature for available evidence on the benefits of statin treatment in distinct patient groups (stratified by age group, primary vs. secondary prevention, diabetes and coronary calcium status). Research articles were identified for examination by searching the MEDLINE database. RCTs, non-randomized retrospective and prospective observational studies, and meta-analyses were assessed primarily involving study populations greater than 10,000. Clinical studies and meta-analyses that treated primary and secondary prevention patients as a single cohort were excluded. Studies of significant interest are summarized in Table 1.

## 6. Efficacy of Statin Treatment in the Elderly

### 6.1. Primary Prevention of ASCVD

Ponce et al. performed a comprehensive meta-analysis of 23 RCTs comprising 60,194 patients aged 65+ [72]. Cohort data was collated for each of five patient groups: ages 65–75, ages 75 and over, patients with diabetes, patients without diabetes, and patients with hypertension. For primary prevention in those with no history of ASCVD, statin use was found to reduce the risk of coronary artery disease [CAD; RR: 0.79, 95% CI: 0.68–0.91] and myocardial infarction (RR: 0.45, 95% CI: 0.31–0.66) relative to placebo in all patient groups. However, Forest plot analysis revealed that statins did not significantly reduce the risk of heart failure (RR: 1.04, 95% CI: 0.80–1.35), all-cause mortality (RR: 0.95, 95% CI: 0.84–1.07), or cardiovascular mortality (RR: 1.01, 95% CI: 0.83–1.24) overall or in any patient group [72]. The one RCT of adults ≥75 reported an increase in all-cause mortality for 40 mg/day pravastatin vs. usual care (HR: 1.34, 95% CI: 0.98–1.84) [73].

Other meta-analyses of primary prevention in the elderly have similarly reported statistically significant RR reductions for ASCVD events but not mortality rates. A meta-analysis of JUPITER and HOPE-3 cohorts aged 65–69 and 70+ by Ridker et al. found non-significant reductions in all-cause mortality [74]. An earlier meta-analysis was performed of 24,674 adults 65+ spanning 8 RCTs as a single cohort (mean age 73 ± 2.9 years) [75]. Statins significantly reduced the rate of myocardial infarction and stroke but not all-cause or cardiovascular mortality. These findings support the conclusion that the benefits of statin therapy are less than maximal in elderly (65+) patients for primary prevention. The weakening dependence of mortality on cholesterol levels at ages over 60 is one explanation for the abatement of statins in extending elderly lifespan [29,67].

### 6.2. Primary Prevention with Diabetes

Limited placebo-controlled RCT data is available for elderly patients with diabetes and very old adults aged 75+ [72]. Ramos et al. conducted a retrospective study of 46,864 participants 75 years and older with no history of ASCVD for a median follow-up of 5.6 years [69]. To prevent survivor bias and covariate measurement bias, a new-user study design was employed in which new statin users were selected for rather than all statin users. A Cox proportional hazards model was used to calculate the HRs of statin use for outcome events. In patients with diabetes aged 75–84, statins were modestly effective in reducing ASCVD [HR: 0.76, 95% CI: 0.65–0.89; 1-year number needed to treat (NNT): 164] and all-cause mortality (HR: 0.84, 95% CI: 0.75–0.94; NNT: 306). The reduction in ASCVD began to lose statistical significance at age 85 and disappeared completely at age 92. The protective effect of statins against all-cause mortality began to lose significance at age 82 in patients with diabetes and disappeared definitively at age 88. Remarkably, in patients without diabetes, statins did not reduce the risk of ASCVD, stroke, CHD, or all-cause mortality for the age groups 75–84 and 85+. The authors went on to point out that under current cholesterol guidelines, most of the population was eligible for statin treatment, having a 10% risk of ASCVD at 10 years [69].

### 6.3. Secondary Prevention of ASCVD

The meta-analysis by Ponce et al. also assessed the effect of statins in the treatment of patients diagnosed with clinically significant ASCVD [72]. RCTs were collected for two patient populations, adults aged 65–75 and patients without diabetes. In contrast to primary prevention, high-certainty evidence was obtained for the use of statins in adults 65 and over for secondary prevention of ASCVD. Compared to placebo, statins significantly decreased the risk of all-cause mortality (RR: 0.80, 95% CI: 0.73–0.89), cardiovascular mortality (RR: 0.68, 95% CI: 0.58–0.79), CAD (RR: 0.68, 95% CI: 0.61–0.77), and myocardial infarction (RR: 0.68, 95% CI: 0.59–0.79). However, evidence was less conclusive for heart failure (RR: 0.79, 95% CI: 0.59–1.06) and stroke (RR: 0.90, 95% CI: 0.79–1.02). No separate data on subgroups, such as those specifically over 75 years of age or patients with type 2 diabetes, was available from the eight trials reviewed.

In 2020, a meta-analysis was performed based on 17 RCTs in elderly patients 65+ for primary or secondary prevention [76]. The substantial statin benefits obtained for secondary prevention closely match the findings of Ponce et al. for ASCVD events and mortality rate. Also in agreement with Ponce et al., in primary prevention statins were observed to significantly reduce the rate of myocardial infarction but not all-cause or cardiovascular mortality [76]. Despite this abundance of evidence from RCTs, two retrospective propensity score-matched studies in narrow populations recently reported reductions in all-cause mortality/ASCVD events in primary prevention adults aged 75+. A study of US veterans (97% men, 91% white) found HRs of 0.75/0.92 [77], while a study of 1278 Koreans found HRs of 0.56/0.59 [78].

## 7. Coronary Artery Calcification for Guiding Treatment Decisions

Robust measures of ASCVD risk are needed to guide treatment decisions in low-risk primary prevention patients. The severity of CAD and plaque formation can be assessed quantitatively based on the level of coronary artery calcification (CAC) in a computed tomography scan. The CAC score has been shown to be a strong independent risk factor for ASCVD and all-cause mortality [79]. A meta-analysis was previously conducted of 49 studies involving 85,000 patients [80]. During a mean follow-up period of 50 months, 0.47% of patients with no CAC suffered a cardiovascular event, compared to 4.1% of patients with CAC, highlighting the metric’s negative predictive power. In a recent retrospective study of 22,346 adults ages 30–49, CAC was present in 34% of patients, of which 7% had CAC >100 [81]. The latter had a 10-fold higher CHD mortality rate compared to those with no CAC. Of the CHD deaths reported in the study period (mean follow-up, 12.7 years), 68% were in individuals who had CAC at baseline.

Mitchell et al. completed a retrospective cohort study of 13,644 consecutive subjects over the age of 18 who underwent CAC scoring, assessing the impact of statin use on first observed MACE in the 9.4-year follow-up period [71]. Outcomes were stratified by baseline CAC score and HRs were determined separately for all statin users (*n* = 6886) and compliant statin users (>50% compliance, *n* = 4415) relative to nonusers. Statin use substantially reduced MACE in those with elevated CAC. HRs for all statin users with CAC values 1–100, 101–400, and 401+ were 0.83 (95% CI: 0.60–1.16), 0.32 (0.21–0.48), and 0.56 (0.34–0.90), respectively. The corresponding HRs for compliant users relative to nonusers were similar for these groups, but not in the case of CAC = 0. Compliant users with no CAC experienced modest reduction (HR: 0.66, 95% CI: 0.49–0.88), but the benefit was abrogated for all users (HR: 1.00, 95% CI: 0.79–1.27). The results illustrate the utility of CAC in identifying patients who will particularly benefit from statin therapy. The 10-year NNT for statin users with a CAC of 1 to 100 was calculated to be 100, while NNT = 12 was obtained for CAC > 100 [71].

A lack of CAC can be used to rule out statin treatment in low-risk individuals. In a recent study of 13 candidate markers of ASCVD including apolipoprotein B, CAC = 0 and CAC ≤ 10 were found to be the strongest negative risk markers in 5805 older adults (mean age 69 y) [82]. Lastly, an inverse association has been observed between CAC density and serum magnesium [83]. Serum magnesium inhibits extracellular calcification, and low serum magnesium is associated with CHD and sudden cardiac death (HRs: 1.36 and 1.54 for lowest quartile) [84]. The 2018 revision of the ACC/AHA guidelines advise that CAC can facilitate treatment decisions when risk status is uncertain [85]. The personalized approach of withholding statins, for example in low-risk elderly patients, has been termed “derisking” [6]. In fact, the stratification of statin benefits by CAC scores can result in much lower NNTs than when using the highest risk categories from a traditional risk calculator, see Refs. [70,71,86] (Table 1). One could argue that a criterion based on CAC score should therefore be added to the four statin benefit groups originally envisioned by the 2013 ACC/AHA guidelines [87]. An alternative approach would be to incorporate CAC into the risk calculator. In a study of U.S. and European adults aged 60-96 (mean: 70 y) without ASCVD, replacing a single variable, patient age, with CAC score was shown to improve traditional models for predicting the rate of incident CHD [88].

The fact that the association of CAC with ASCVD risk remains significant in elderly patients contrasts with many traditional risk factors [82,88]. The hazard ratio of BMI for myocardial infarction has been shown to decline rapidly from age 50 to age 70, and similarly for the association of systolic blood pressure with ischemic stroke and heart failure [89]. In the same study, LDL-C but not HDL-C remained associated with myocardial infarction up to the study endpoint of age 82.

## 8. Coenzyme Q and Muscle Function: Role for Supplementation

The mevalonate biosynthetic pathway is responsible for endogenous production of both cholesterol and ubiquinone, or coenzyme Q_10_ (CoQ). CoQ is an important antioxidant and functions as an electron carrier in the mitochondrion responsible for cell respiration. Endogenous levels of CoQ in plasma and in various organs decline with age [90,91], and decreased CoQ has been implicated in age-related diseases [92]. A population study in Israel recently associated CoQ supplementation with reduced odds of SARS-CoV-2 hospitalization (OR: 0.185, 95% CI: 0.06–0.46) [93]. Earlier studies observed that low levels of plasma CoQ associate with increased mortality in chronic heart failure (CHF) in the elderly [94,95]. Long-term dietary supplementation with 300 mg/d CoQ in addition to standard CHF therapy has since been shown to reduce MACE as well as cardiovascular and all-cause mortality in RCTs of older adults [96,97]. Meta-analysis of 14 RCTs found that CoQ supplementation significantly decreased mortality (RR: 0.69, 95% CI: 0.50–95) and increased exercise capacity in CHF patients compared to placebo [68].

Statin drugs are inhibitors of the enzyme HMG-CoA reductase, which catalyzes the rate-limiting step of converting HMG-CoA into mevalonate. Statin use has been demonstrated to decrease plasma levels of CoQ in a dose-dependent fashion [94,98,99]. The inverse association between CoQ levels and heart failure could help explain why statins alone do not decrease the risk of heart failure in elderly patients without ASCVD, and offer non-significant benefit in secondary prevention (RR: 0.79, 95% CI: 0.59–1.06) [72].

Mitochondrial function is essential for muscle fitness and maintaining health in old age. A translational research study by Schirris et al. elucidated the mechanism of statin-induced myopathy [100]. Using a myoblast cell line, the cyclic lactone forms of the seven statins were found to more potently induce cytotoxicity and reduce respiration and mitochondrial ATP production compared to their carboxylic acid equilibrium forms. The off-target mechanism of action involved up to 84% inhibition of mitochondrial electron transport chain complex III (CIII). CIII is a ubiquinol oxidase that oxidizes CoQH_2_ into CoQ, transferring its electrons to complex IV in order to reduce molecular oxygen. In muscle biopsies of 37 patients with statin-induced myopathy, CIII activity and ATP production decreased by up to 18% compared to healthy controls [100]. Analogous to an earlier cell study with simvastatin [101], respiratory inhibition could be prevented by increasing the endogenous pool of CoQH_2_, which competes for the same binding site on CIII as the statin lactone. In a recent study of 571 adults taking atorvastatin, cigarette smoking was found to increase lactonization via enzyme induction [102]. The study also associated increased age, low BMI, proton pump inhibitors, and loop diuretics with increased levels of exposure to atorvastatin, factors thought to increase the risk of statin-associated myotoxicity [102,103].

The observations on muscle function and heart failure would seem to suggest that CoQ should be coadministered with long-term statin therapy. Well-designed RCTs demonstrate CoQ to be safe and effective in treating a number of human diseases such as primary CoQ deficiency syndrome, a rare mitochondrial disease, with a highest possible dose of 1200 mg/d [92,104]. Small clinical trials of statin and CoQ coadministration provided some early evidence of reducing statin-associated myopathy [105,106]. A 2018 meta-analysis has since identified 12 RCTs involving 575 patients [107]. Compared with placebo, CoQ supplementation significantly decreased statin-associated muscle pain, weakness (*P* = 0.006), cramps, and tiredness (*P* < 0.001). Despite myalgia being the most common cause of statin intolerance and medication nonadherence, current clinical guidelines have yet to recommend routine use of CoQ in statin users [40,85]. The reduced form of CoQ, ubiquinol (CoQH_2_), is now being explored as a superior supplement and antioxidant. The ratio of CoQH_2_ to CoQ has been shown to decrease with age and is reduced in patients with geriatric diseases, indicative of systemic oxidative stress [108]. Ubiquinol reverses statin inhibition of CIII [100], and has superior bioavailability compared to its oxidized form ubiquinone [109,110,111].

Selenium is an essential antioxidant that is needed for enzymatic reduction of CoQ to its active form, ubiquinol [112]. The soil content of selenium is low in Europe compared to the U.S. An RCT was conducted of 443 healthy Swedes aged 70–88 (20% statin users) whom were given placebo or CoQ capsules 100 mg B.I.D. + selenium tablets 200 μg/d for 4 years [113]. Follow-up results were obtained for a period of up to 10 years after intervention, and Cox regression analysis was used to measure the cardiovascular mortality risk [114]. Significant reductions were observed for the treatment group in cardiovascular mortality (HR: 0.51, 95% CI: 0.36–0.74, *P* = 0.0003) and plasma NT-proBNP, a biomarker for heart failure [113,114].

Ubiquinol itself is now produced as a supplement. A small trial was conducted using ubiquinol 600 mg/d, but it failed to demonstrate an effect on myalgia symptoms [115]. Taylor et al. first used an eight-week double-blind crossover trial to eliminate patients without confirmed statin-associated myalgia. Only 41 of 120 subjects, who experienced pain on simvastatin and not placebo, and whose pain resolved within four weeks off treatment, were chosen for inclusion in their subsequent analysis. Other limitations in the small trial were that a moderate-intensity statin was used and only 32 patients completed the second crossover treatment and received CoQH_2_. The authors estimated a sample of *n* = 720 would be needed to prove ubiquinol reduces statin myalgia [115]. Large scale RCTs will ultimately be needed to confirm the protective effect of ubiquinol in statin users. Research in this area has also been hampered to some extent by the need for an objective means of diagnosing myalgia, myopathy, and myositis [116].

## 9. Primary Prevention in Middle-Age Adults

Given the relative weakness of evidence for the use of statins in primary prevention of ASCVD, further research is needed to assess the efficacy of statins in middle-aged adults. Unfortunately, RCTs and meta-analyses of statins across age groups have typically incorporated participants with a prior history of ASCVD [10,117,118]. One meta-analysis was performed on 11 RCTs involving 65,229 high-risk patients in the use of primary prevention [119]. Statins did not reduce all-cause mortality (RR: 0.91, 95% CI: 0.83–1.01). Using meta-regression, the baseline age of each of the 11 study cohorts was able to explain 66% of the variation in mortality rate, however. This finding illustrates how confounding variables can potentially influence our analysis and interpretation of the whole of evidence from clinical trials. An earlier meta-analysis was conducted of 20 RCTs in which >50% of adult participants had no history of CHD [120]. In a total of 65,261 patients, statins exhibited small reductions in MACE (RR: 0.85, 95% CI: 0.77–0.95), ASCVD deaths (RR: 0.89, 95% CI: 0.81–0.98), and all-cause mortality (RR: 0.93, 95% CI: 0.87–0.99).

Retrospective analysis of an electronic patient database allows one to examine large population-level cohorts incorporating specific groups often excluded from RCTs (e.g., poor statin tolerance), thus evaluating treatment efficacy in real clinical conditions. While statin treatment guidelines emphasize maximal relative risk reductions, a recent Spanish population study by Garcia-Gil et al. determined the 5-year NNT for adults aged 35–74 in primary prevention [70]. A total of 617,850 patient records were analyzed from the SIDIAP database. Statins were found to decrease ASCVD events according to a patient’s 10-year CHD risk category (Table 1). In statin users with >70% adherence, statins decreased ASCVD in individuals with 10-year risk categories of 7.5–10% (HR: 0.70, 95% CI: 0.54–0.92; NNT = 75) and 10–20% (HR: 0.74, 95% CI: 0.61–0.90; NNT = 62). All-cause mortality reduction was significant only for individuals with 10–20% risk (HR: 0.73, 95% CI: 0.59–0.91; NNT = 69). Regardless of CHD risk, no clinically relevant effect size was found for ASCVD or all-cause mortality in patients with low adherence to therapy [70]. Medication nonadherence is a common problem due to statin intolerance, and the fraction of study participants achieving adequate reductions in LDL-C is estimated to range from 20% to 64% [121].

## 10. Effect of Lipid-Lowering Medication on Lipoprotein Subfractions

Further research is needed to examine the impact of lipid-lowering medications on the relative proportions of atherogenic lipoprotein particles in the blood. A covalently modified form of LDL, lipoprotein(a) [Lp(a)], is considerably atherogenic and is observed to be elevated three-fold in CHD patients [122]. A meta-analysis of seven RCTs involving 29,069 adults revealed statins reduced LDL-C by a mean of 39% (95% CI: 35–43), while not significantly changing Lp(a) [123]. Baseline and on-statin levels of Lp(a) were associated with ASCVD risk, with HRs for ≥50 mg/dL (vs. <15 mg/dL) = 1.31 (95% CI: 1.08–1.58) and 1.43 (95% CI: 1.15–1.76), respectively. In contrast with statins, PCSK9 inhibitors are a new class of monoclonal antibody therapy developed to lower LDL-C. Pooled analysis of 3,278 patients from 10 clinical trials demonstrated that biweekly evolocumab treatment reduced Lp(a) by 25% [124]. Other studies have found statins to slightly increase the relative proportion of atherogenic Lp(a) and sdLDL [125,126].

Ongoing research efforts are examining the role of cholesterol in triglyceride-rich lipoproteins (VLDL, IDL, chylomicron remnants), termed remnant cholesterol. In a Danish population study, 32%, 35%, and 32% of nonfasting plasma cholesterol was contained in remnant lipoproteins, LDL, and HDL, respectively [127]. Remnant cholesterol has been shown to be one of the best lipid markers for predicting myocardial infarction and can be easily calculated [128,129,130]. Relative to the lowering effect on LDL-C, remnant cholesterol was observed to be reduced by 80% through either statin treatment or genetic inhibition of HMG-CoA reductase in a metabolomic profiling study [131]. Icosapent ethyl has been shown to reduce remnant cholesterol by 21% compared to placebo and by 57% when taken in conjunction with statin therapy [130]. The REDUCE-IT trial examined the role of icosapent ethyl in reducing the residual cardiovascular risk not addressed by statin treatment [132]. Patients were eligible who had been receiving statin therapy, had triglyceride and LDL-C levels of 135–499 (median, 216) and 41–100 (median, 74) mg/dL, respectively, and had established ASCVD or diabetes. Adjuvant treatment with icosapent ethyl was observed to decrease triglycerides by 18% and reduce ASCVD events (HR: 0.75, 95% CI: 0.68–0.83) compared to statin treatment alone. Other RCTs have associated triglyceride levels with residual coronary risk [133]. Of note, one fifth of U.S. adults with diabetes have residual hypertriglyceridemia, despite their LDL-C being well-controlled by statins [134].

Recent guidelines and consensus papers have promoted non-HDL (TC minus HDL-C) for ASCVD prediction, which is equivalent to LDL and remnant cholesterol combined [40], see also Refs. 11–13 in [129]. Earlier analysis revealed a log-linear association of non-HDL and HDL-C with CHD risk in 302,430 Europeans and North Americans [41]. Non-HDL correlated strongly with apolipoprotein B, and the hazard ratios were as least as strong when computed for individuals who did not fast compared to those who did, further speaking to their ease of measurement. After adjusting for HDL-C and non-HDL, the association between plasma triglyceride and CHD was abolished [41]. This has led some to conclude that triglyceride is largely a surrogate clinical marker, and that cholesterol is the principal lipid deposit within atherosclerotic plaque [129]. The PURE study recently estimated the population-attributable fractions of ASCVD for 14 behavioral and metabolic risk factors [64]. Non-HDL was the strongest lipid risk factor for ASCVD (HR: 1.31 highest/lowest tertile, 95% CI: 1.21–1.41).

## 11. Perspective

Given that ASCVD is a cardiometabolic disorder, an important dimension of statin treatment is the role of habitual diet. Analysis of 27,886 U.S. adults from 1999 to 2010 indicated that caloric and fat intake and BMI increased among statin users by 10%, 14% and +1.3, respectively, while not changing in nonusers [135]. The importance of diet and nutrition must be emphasized during patient medication counseling. Trimethylamine *N*-oxide, a metabolite of animal products, has been implicated in arterial plaque development [136], and plasma levels are a strong risk marker for MACE and all-cause mortality (pooled RRs: 1.62 and 1.63) [137]. A Mediterranean-style diet low in meat and dairy products has been reported to reduce the relative risk of MACE and cardiovascular mortality by 30% [138,139]. New lines of research suggest a synergistic interaction between statin treatment and dietary improvement [140]. In a prospective observational study of secondary prevention in southern Italy (mean age 67.7 ± 10), statins decreased all-cause and cardiovascular mortality only in patients with an average or high Mediterranean diet score, and by 50% compared to the control group of non-statin users with low diet score [141]. Rather than furthering improving blood lipids, a Mediterranean diet was found to reduce low-grade inflammation in the presence of statins, shedding light on the mechanism of interaction between diet and mortality benefit.

Statins are increasingly prescribed using a treat-all approach to prevent ASCVD. The broadening of diagnostic criteria in recent decades has led to their use in low-risk and elderly individuals, dramatically altering the absolute risk reduction and NNT obtained for MACE and mortality outcomes [2,7,10,70]. Current clinical guidelines emphasize identifying a patient’s family history of dyslipidemia. After excluding familial hypercholesterolemia, however, LDL-C or triglycerides > 90th population percentile associate similarly with CAD risk regardless of family history [142]. Maximal reduction in LDL-C has long been the target of statin therapy. The 2013 ACC/AHA guidelines referenced an oft-cited 28% reduction in RR per mmol/L reduction in LDL-C [87]. In contrast, a recent meta-analysis of intensive statin therapy for primary and secondary prevention combined found a modest RR of 14% for adults whose baseline LDL-C was ≥100 mg/dL [118].

Despite widespread use, current evidence on the benefit effect size does not support the use of statins for primary prevention in very old adults, in whom the significance of adverse effects is amplified [6]. The U.S. Preventive Services Task Force concluded that the evidence does not support statin therapy for primary prevention of ASCVD in adults over 75 [61], consistent with the evidence presented here for octogenarians. The AGREE II instrument was used to evaluate 33 international guidelines for ASCVD prevention [143]. Only 18 guidelines were found to provide instructions for discontinuation of statins, which were related to general intolerance or poor health rather than age status. Their findings underscore the lack of a deprescribing tradition and the growing reliance of practice guidelines on low-level evidence and expert opinion [144]. One French population study of 120,173 statin users aged 75 or older did find an increased risk of ASCVD upon statin discontinuation (RR: 1.33, 95% CI: 1.18–1.50), however mortality rates were not assessed [145].

## 12. Conclusions

To predict the benefits and harms of lipid-lowering medications in select individuals, access to full clinical trial data and new trials incorporating specific patient populations are needed [2,10]. Outcomes data available for specific patient groups and improvements in risk assessment enable healthcare providers to better to balance the risks and benefits of statin therapy (Table 2). Coenzyme Q is an effective therapeutic adjuvant for CHF, and supplement formulations may prove of benefit to older adults experiencing statin-associated myopathy. Better metrics now exist for predicting ASCVD risk compared to calculated LDL-C, such as the CAC score, which can be used to rule out treatment in low-risk cases for young and old adults alike. Ultimately, hyper-individualized approaches employing more accurate risk factors and tools will identify the patients that benefit most from lipid-lowering therapy.

## Figures and Tables

**Table 1:** Large-scale clinical studies of significant interest: role of lipid levels, treatment intervention, and biomarkers in distinct patient groups.

Study/Design	Population/Size	Intervention/Outcome Assessed	Major Findings
**Emerging Risk Factors Collaboration, 2009 [41]** 68 long-term prospective studies from ERFC with complete data.	302,430 adults in 21 countries (Europe and North America) with no ASCVD at baseline. 2.79 million person-years	Prediction of CHD events (myocardial infarction, stroke, or CHD deaths): adjusted HRs for 1-standard deviation higher baseline values.	Non-HDL and HDL were log-linear risk factors, but triglycerides were not an independent risk factor after adjustment (HRs: 1.50, 0.78 and 0.99).
**Ravnskov, 2016 [29]** Meta-analysis	19 studies including 30 cohorts with a total of 68,094 adults age 60 and over.	Association of baseline LDL-C with all-cause mortality (irrespective of statin treatment).	Mortality decreased with increasing LDL-C quartile in 92% of participants (mean Q4 HR: 0.54).
**Lei, 2017 [68]** Meta-analysis	14 randomized trials involving 2149 patients with heart failure.	Effect of coenzyme Q supplementation on heart failure outcomes.	Coenzyme Q decreased mortality (RR: 0.69, 95% CI: 0.50–0.95).
**Ramos, 2018 [69]** Retrospective cohort: SIDIAP database; new-user design	46,864 new and non-statin users in Spain aged 75 and older with no history of ASCVD.	New statin use stratified by diabetes and two age groups: 75–84, 85+; mortality/ASCVD events.	Statins reduced ASCVD in diabetes patients (HRs: 0.76, 0.82). No ASCVD reduction in patients without diabetes (HRs: 0.94, 1.00).
**Garcia-Gil, 2018 [70]** Retrospective cohort: SIDIAP database	617,850 primary prevention patients in Spain aged 35–74 (new users, 80% with moderate intensity statin).	ASCVD events (myocardial infarction, ischemic stroke) and all-cause mortality stratified by 10-year CHD risk categories.	5-year NNTs for ASCVD: 470, 204, 75, and 62 for <5%, 5–7.5%, 7.5–10%, and 10–20% risk categories.
**Mitchell, 2018 [71]** Retrospective cohort	13,644 military subjects over 18 with no prior ASCVD; 9.4-year median follow-up.	Effect of statin use versus non-use on first MACE, stratified across six CAC patient groups.	HRs for CAC = 0, 1–100, 101–400, and >400 were 1.0, 0.83, 0.32, and 0.56 (statin use vs. non-use).
**Yi, 2019 [67]** Prospective cohort: KOMERIT: Korean Metabolic Risk Factor	12.8 million Korean adults; age groups: 18–34, 35–44, 45–54, 55–64, 65–74, 75–99.	Relationship between TC and all-cause mortality for 12 age-sex groups.	HR was a U-shaped function of TC in all 12 groups. HRs approached 1.0 for TC: 185–275 in elderly adults.
**Ponce, 2019 [72]** Meta-analysis	23 randomized trials involving 60,194 adults 65 and older.	Statins for primary versus secondary prevention; all-cause and cardiovascular mortality/ASCVD events.	Strong evidence supporting statins for secondary but not primary prevention.
**Yusuf, 2020 [64]** Prospective cohort: PURE (Prospective Urban Rural Epidemiology)	155,722 adults aged 35–70 without ASCVD followed for 9.5 years; 21 low and high-income countries across 5 continents.	Effects of 14 modifiable behavioral and metabolic risk factors on ASCVD events and all-cause mortality.	70% of ASCVD events and deaths were attributable to the 14 risk factors, especially hypertension (22%) and high non-HDL-C (8%).

Abbreviations: ASCVD, atherosclerotic cardiovascular disease; CAC, coronary artery calcification; CHD, coronary heart disease; ERFC, Emerging Risk Factors Collaboration; HDL, high-density lipoprotein cholesterol; HR, hazard ratio; LDL-C, low-density lipoprotein cholesterol; MACE, major adverse cardiovascular event; NNT, number needed to treat; Q4, highest quartile; SIDIAP, Information System for the Development of Research in Primary Care; TC, total cholesterol.

**Table 2:** Conclusions, statin benefit groups, and robust markers of cardiovascular risk.

Cardiovascular Risk Factors	Contraindications/Adverse Effects
Prior history of ASCVD: statins reduce mortality and ASCVD in secondary prevention [72,76].	Lack of benefit in primary prevention over age 75 without diabetes [69,72]. Derisk at age 65 if no risk factors [6,62].
CAC = 0 and ≤10 are negative risk markers in old and young adults [81,82]. CAC > 100 may be considered a statin benefit group: 10-year NNT = 12 [71].	Myopathy/osteoporosis/new-onset diabetes at high doses; avoid canagliflozin (see Introduction) [11].
Statins modestly reduce ASCVD in primary prevention diabetes patients aged 75–84: 1-year NNT = 164 [69].	Coenzyme Q supplementation improves health outcomes [68,107,114].
Non-HDL (LDL particle number + remnant cholesterol) is more predictive than LDL-C_F_ [64,129].	Residual cardiovascular risk persists after LDL-C controlled by statins alone [133].
2013 pooled cohort equations overestimate 10-year ASCVD risk: increase 7.5% risk threshold to 10% for primary prevention [6,55,61].	Healthy diet found necessary for mortality benefit of statins in secondary prevention [141].

Abbreviation: LDL-C_F_, traditionally calculated low-density lipoprotein cholesterol (Friedewald equation).
